# Crosstalk between high-density lipoproteins and endothelial cells in health and disease: Insights into sex-dependent modulation

**DOI:** 10.3389/fcvm.2022.989428

**Published:** 2022-10-06

**Authors:** Elisa Dietrich, Anne Jomard, Elena Osto

**Affiliations:** ^1^Institute for Clinical Chemistry, University of Zurich and University Hospital Zurich, Zurich, Switzerland; ^2^Department of Cardiology, Heart Center, University Hospital Zurich, Zurich, Switzerland

**Keywords:** HDL, endothelial cells, sex differences, cardiovascular disease, HDL-endothelial crosstalk

## Abstract

Atherosclerotic cardiovascular disease is the leading cause of death worldwide. Intense research in vascular biology has advanced our knowledge of molecular mechanisms of its onset and progression until complications; however, several aspects of the patho-physiology of atherosclerosis remain to be further elucidated. Endothelial cell homeostasis is fundamental to prevent atherosclerosis as the appearance of endothelial cell dysfunction is considered the first pro-atherosclerotic vascular modification. Physiologically, high density lipoproteins (HDLs) exert protective actions for vessels and in particular for ECs. Indeed, HDLs promote endothelial-dependent vasorelaxation, contribute to the regulation of vascular lipid metabolism, and have immune-modulatory, anti-inflammatory and anti-oxidative properties. Sex- and gender-dependent differences are increasingly recognized as important, although not fully elucidated, factors in cardiovascular health and disease patho-physiology. In this review, we highlight the importance of sex hormones and sex-specific gene expression in the regulation of HDL and EC cross-talk and their contribution to cardiovascular disease.

## Introduction

The relationship between high-density lipoproteins (HDLs) and cardiovascular disease (CVD) is a topic of intense investigation since decades (1).

Epidemiological studies have shown a correlation between low levels of HDL-cholesterol (HDL-C) and increased incidence of CVD (2). Indeed, a U-shape correlation has been recently reported whereby both low (< 50 mg/dl in women and < 40 mg/dl in men; 0.8 and 1.3 mmol/L, respectively) and high (>80 to 90 mg/dl; >2.3 mmol/L) levels of HDL have been associated to increased all-cause and CV mortality in both men and women without previous CVD (3–5).

Increasing evidence suggests that rather than cholesterol levels present on HDL, HDL particle number, lipid and protein composition play a key protective role in reducing CVD risk (6–8). HDL particle composition directly influences HDL vaso-protective functions (i.e. reverse cholesterol transport (RCT), nitric oxide (NO) production from endothelial cells (ECs), anti-oxidative and anti-inflammatory properties).

ECs are a physical barrier between blood and body tissues, which act as gatekeepers of cardiovascular homeostasis. Indeed, EC-released vasoactive substances (in particular NO) regulate hemostasis, control vascular permeability and modulate both acute and chronic immune responses to injuries (9). In light of its strong vasodilatory, anti-inflammatory and anti-oxidative properties, NO plays a central role in the maintenance of vascular health (10). Reduction in NO bioavailability is the hallmark of endothelial cell dysfunction (ECD), which in turn favors atherosclerosis (11).

Sex-related inter-individual variability (hormonal levels, hormone therapies, gene expression profiles etc.) can influence CVD risk by acting on both HDLs and ECs (12–14).

Increasing evidence suggests that sexual hormone levels—in particular testosterone and estradiol—and sex-specific cellular gene expression profile can influence not only HDL-C levels but also HDL subclasses and function. Indeed, men display reduced levels of HDL-C and a more pro-atherogenic phenotype compared to women (15–17).

Furthermore, estrogens are well-recognized EC protective molecules, able to stimulate NO production, EC growth and wound healing mechanisms (18, 19). Of note, differences in gene expression profile between female and male ECs appear to influence EC susceptibility to insults, with the activation in female ECs of more efficient stress-response mechanisms compared to male ECs (20, 21). These differences could explain, at least in part, why pre-menopausal women have lesser CVD risk than age-matched men and could give useful hints for personalized therapy development.

In this Review, we mainly focused on the influence of sex-specific factors on both HDL and EC function and how sex-dependent differences modulating HDL-EC cross- talk may contribute to the CV protection of pre-menopausal women compared to age-matched men (22–24). Indeed, sex closely interacts with gender in the development of atherosclerosis therefore, although not systematically addressed, some gender-specific aspects (i.e., pertaining to the socio-economic and cultural sphere) have been also mentioned in case of their known influence on HDL and EC function and potential CV patho-physiological impact (23–26).

## HDL-targeting drugs: The failure of cholesteryl ester transfer protein inhibitors

The concept that HDL is the “good cholesterol” first originated from the Framingham Heart Study, which showed strong inverse association between HDL-C and coronary heart disease (CHD) (27). However, this concept has been challenged by results of following clinical trials in which cholesteryl ester transfer protein (CETP) inhibitors, despite raising HDL-C levels, failed to reduce CV morbidity and mortality. These results suggested that beyond the simple increase of HDL-C plasma levels, the modulation of HDL composition could be more important to achieve cardiovascular benefits (28–30). CETP is a plasma protein that transfers cholesteryl ester from HDL to apolipoprotein B (ApoB)-containing lipoproteins in exchange for triglyceride (TG). The inhibition of CETP leads to higher cholesterol levels in HDLs. Indeed, species lacking CETP and patients with CETP deficiency are characterized by increased HDL-C levels and reduced risk for CVD (31–34). In the Investigation of Lipid Level Management to Understand its Impact in Atherosclerotic Events (ILLUMINATE) trial, the CETP inhibitor Torcetrapib increased HDL-C levels as expected, but this increase was not paralleled by decreased CHD and the trial was stopped due to elevated risk of cardiac and death events (35).

In line with the notion that HDL-C alone may not be a reliable marker of the cardio-protective quality of HDLs, it has been recently shown in a sex-mixed pool of patients that CETP inhibitors, Torcetrapib and Evacetrapib, not only increased HDL-C but also enhanced the concomitant content of apoC3/apoE in HDLs. These two proteins rendered HDLs dysfunctional and were associated with higher CHD (36). Different CETP inhibitors, such as Dalcetrapib and Anacetrapib slightly reduced CHD risk, although this effect could have been influenced by the concomitant reduction in non-HDL-C in treated patients (37–42).

Genetic polymorphisms associated with increased HDL-C levels also did not influence the risk score for myocardial infarction (1, 43). Population studies carried out in Copenhagen highlighted a dramatic enhancement of CHD risk in women with CETP deficiency, in spite of the elevated HDL-C levels (44). Furthermore, as result of rare genetic variants on scavenger receptor BI (SR-BI) gene and reduced ability of HDLs to deliver cholesterol to the liver, the consequent increased HDL-C levels were linked to higher rather than lower risk of CHD risk in both men and women (45, 46). Indeed, the increased cholesterol in HDL in these specific circumstances was linked to an impaired HDL-mediated RCT. Taken together; this evidence questioned the rationale of using CETP inhibitors as treatment for CVD and highlighted the need for a better characterization of the complexity of HDL, in particular focusing on HDL composition as key determinant of function in health and disease.

## Sex- and age-related differences in HDL measurements

**Figure 1:**
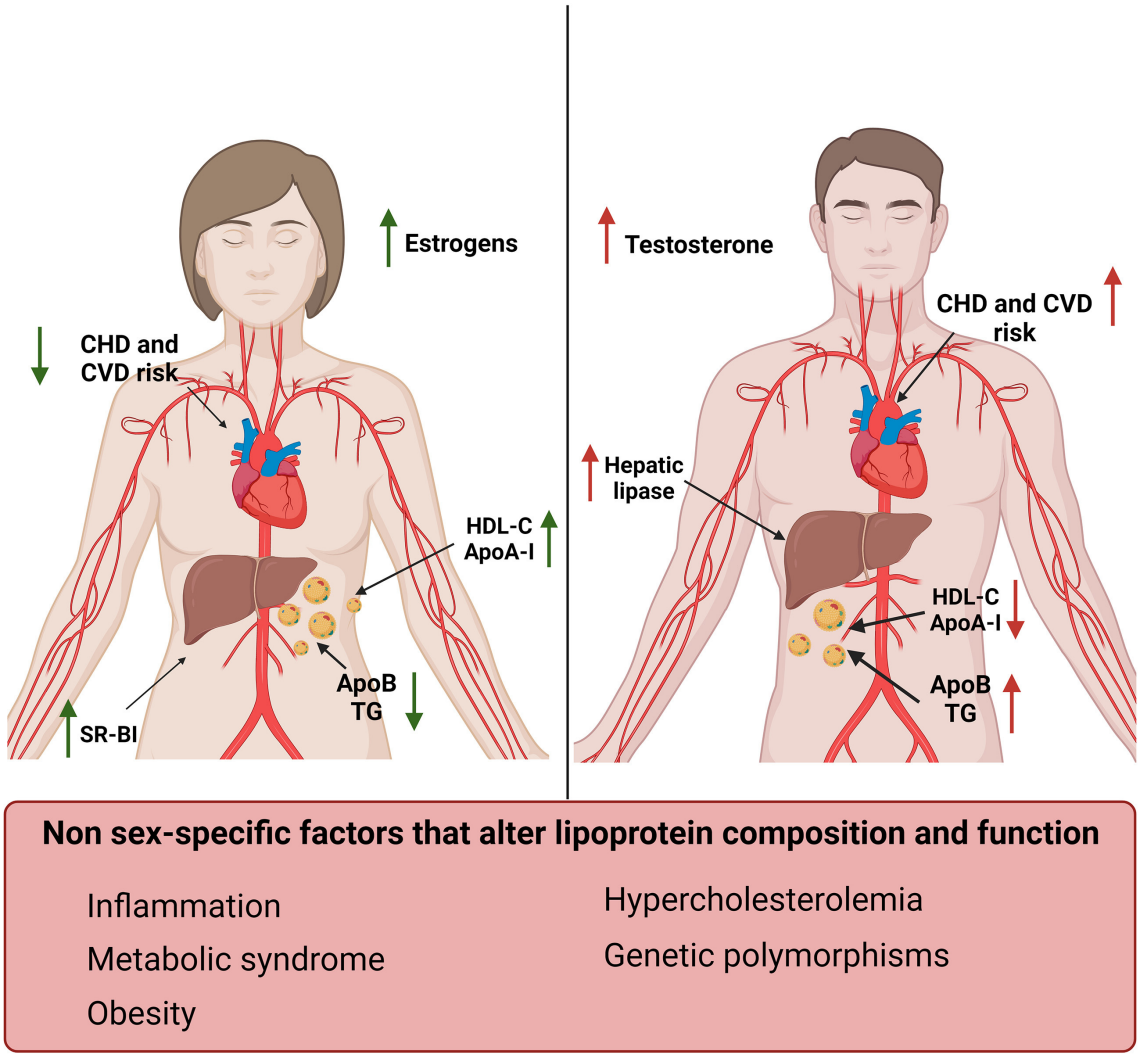
Sex-specific differences in lipoproteins and their effect on cardiovascular risk factors. Women **(left)** display increased levels of HDL-C and ApoA-I and reduced levels of TG and ApoB compared to men **(right)** (12, 49–52). Men also display increased levels of hepatic lipase compared to women, which in turn have higher SR-BI expression levels. These differences correlate with estrogens and testosterone levels and result in a reduced risk for CVD and CHD in women compared to men (53). Sex-independent factors associated with increased risk for CVD and changes in lipid profile have also been reported in this figure **(bottom)**.

HDL-C is commonly used as a predictor marker for CVD risk, as reported in SCORE (Systematic Coronary Risk Evaluation) risk charts and the ASCVD Pooled Cohort Equations (47, 48). So far, reference values for lipid profiles, including HDL, used in clinical practice are the same for both men and women, despite growing evidence on the influence of sex differences in the discriminative performance of CVD risk scores. Indeed, pre-menopausal women have higher HDL-C levels and lower risk of CVD compared to men (Figure 1) (12, 49–51). Phases of menstrual cycle may influence lipid profile. While post-prandial serum TG were higher in women during the follicular compared to the luteal phase of menstrual cycle, HDL and ApoB levels were stable in both phases (54). In another study, a significant decrease in the mean levels of TC, LDL-C, TC/HDL-C, LDL/HDL and TG/HDL was observed in the luteal compared to the follicular phase of menstrual cycle (55). Some studies suggested assessing female parameters during the follicular phase of menstrual cycle could help to minimize differences due to sexual hormones fluctuations (55, 56).

Patient age is another important factor influencing sex-dependent differences, since HDL-C levels can vary during individual lifetime. Healthy pre-pubertal children had high levels of HDL-C independently from their sex (56). HDL-C levels then drastically decreased in boys after puberty (~45 mg/dL/1.16 mmol/L), while remaining higher in girls (~55 mg/dL/1.42 mmol/L) (57). These differences were lost in post- menopausal women, independently from menopausal age (58, 59).

Hormonal therapies can also alter lipid parameters. Transgender men (i.e., female to male) displayed a clear reduction in HDL compared to women, but higher levels than cis-gender men (25, 60).

These sex- and age-dependent differences need to be taken into account when HDL-C levels are used as a CVD prognostic marker. Moreover, when considering the relationship between high HDL-C levels and increased all cause and CV mortality, relevant factors to be evaluated are sex differences together with the presence of CVD and other comorbidities. In fact, the increased cardiovascular risk associated with high HDL-C initially reported in a sex-mixed pool of patients without previous cardiovascular conditions by the CANHEART Study and others (3, 5) has not been confirmed afterwards in women with hypertension (61). Another study analyzed six community-based cohorts and showed that in men the inverse linear association between HDL-C and CHD events has a broader span compared to women. For HDL-C values >90 mg/dL (>2.33 mmol/L) in men and HDL-C values >75 mg/dL (>1.94 mmol/L) in women, the association between HDL-C and CHD events reached a plateau with no further reductions in CHD risk (62).

All-cause mortality in healthy, smoking, middle-aged (50–59 years) and older (>60 years) Finnish men was positively associated with HDL-C in the middle-aged group, while there was a U-shaped pattern in older men. Of note, the middle-aged group had a higher reported alcohol intake than the older individuals. Moreover, alcohol- and violence-related mortality was strongly positively associated with HDL-C specifically in the middle age group (63). Thus, alcohol may have influenced the association of HDL-C and mortality through its HDL raising effect and being a risk factor for behavioral-related non-natural as well as alcohol-related deaths beyond coronary disease, such as cancer, cardiomyopathy, stroke (5, 63).

## Insights into sex-dependent and independent differences in HDL structure and composition

HDLs are heterogeneous lipoproteins formed by a cholesterol ester and TG enriched hydrophobic core and a surface lipid bilayer containing mainly free cholesterol, phospholipids and various proteins (6, 64). The biogenesis of HDLs starts from the synthesis and secretion of apolipoprotein- AI (ApoA-I) in the liver and intestine (65). The interaction between secreted ApoA-I and cell membrane protein ATP-binding cassette transporter A1 (ABCA1), expressed by hepatocytes and enterocytes (66), allows the acquisition of lipids and formation of nascent HDLs. Nascent HDLs are converted into mature particles *via* cholesterol esterification performed by lecithin-cholesterol-acyl transferase (LCAT) (67). Endothelial lipase and hepatic lipase are involved in the lipolysis of phospholipids and TGs in HDLs, leading to smaller HDL particles (68). Phospholipid transfer protein further exchanges lipids between HDLs (69). HDL clearance is orchestrated by SR-BI and CETP, which regulate the transfer of cholesteryl-ester from HDLs to the liver and the exchange of ApoB-containing lipoproteins with TGs (70).

Women have increased HDL-C and ApoA-I levels and lower ApoB compared to men. These sex-related differences in plasma lipoproteins start to be evident during puberty, in concomitance with the increase in testosterone in males and estrogens in females (Figure 1) (52).

Estrogens increased ApoA-I expression in the liver and HDL-C levels in pre-menopausal women by modulating the expression of SR-BI and hepatic lipase (71–73). On the contrary, testosterone administration enhanced hepatic lipase activity, increasing HDL catabolism (Figure 1) (52). Androgen therapy was also associated with an unfavorable shift toward an atherogenic lipid profile characterized by reduced ApoA-I and increased apo-B levels in men (74). Suppression of androgens in men, in fact, leaded to an increase in HDL-C, ApoA-I and reduced ApoB levels (75). It has also been shown that hyperandrogenism, which is a common feature of polycystic ovary syndrome, was associated with lower HDL-C levels and dyslipidemia (76, 77).

Differences in lipid profile have also been associated with sex-specific gene expression profile. The KDM6A gene encodes for a histone-demethylase protein highly expressed in the female liver and its expression levels positively correlated with HDL-C (78, 79). In turn, KDM6A silencing in hepatocytes lead to downregulation of genes regulating HDL-C levels (13).

Single nucleotide polymorphisms (SNPs) on the CETP gene have been associated with higher HDL-C and ApoA-I levels (80, 81). TaqIB is the most common SNP variant of the CETP gene and the TaqIB genotype can be expressed as either dominant B1B1 homozygote, B1B2 heterozygote or recessive B2B2. In particular, B2B2 carriers had higher HDL-C plasma levels and 20% lower risk of CHD vs. the B1B1 carriers (82). Of note, the increase in HDL-C levels in CETP-TaqIB, B2B2 carriers seemed to be independent from sexual hormones (81) and was lost in obesity and type 2 diabetes (T2D). Indeed, other CETP SNP variants in both sexes were not associated with HDL-C levels nor with metabolic syndrome and obesity (83). A 16% increase in HDL-C levels has been reported in men with B2 TaqIB variant affected by T2D compared with those homozygous for the B1 allele (83).

ApoE, encoded by APOE gene, is the major ligand for clearance of TG-rich lipoproteins and has anti-atherogenic function (84, 85). APOE-e2 polymorphism has a sex-specific effect on lipid profile and has been associated with high HDL-C levels in woman and increased TG levels in men (86). There are no sex-differences reported for ApoE isoform 4 in the context of CVD risk, while the ApoE4 allele seems to confer a memory advantage in midlife men and an increased risk of Alzheimer in women (87, 88).

HDL-associated LCAT increased mass concentration and higher LCAT activity have been correlated with CHD risk in women but not in men (89, 90). However, mechanisms of the sex-specific association of LCAT and CV risk need further investigation given the conflicting results so far available, for instance in patients with sickle cell anemia and proteinuria where a less pronounced reduction of LCAT activity in women compared to men has been considered protective against accelerated kidney disease progression in this patient population (91). Moreover, LCAT deficiency led to the development of spontaneous atherosclerotic lesions similarly in aged male and female mice (92) and a female specific protection against diet-induced obesity and insulin resistance has been described in mice with combined LCAT and LDL receptor deficiency (93).

Inflammation decreases HDL-C levels and altered HDL composition in a sex-independent manner (Figure 1) (94, 95). Changes in the HDL-associated lipids include a decrease in cholesterol ester and an increase in free cholesterol, TG, free fatty acids and ceramide-enriched lipoproteins. Dysfunctional HDLs show marked alterations in protein composition and become pro-atherogenic. These changes include an increase of serum amyloid A (SAA), a decrease apoA-I but also variations in enzymes and transfer proteins, such as LCAT, CETP, PON-1, and apolipoprotein-M (apoM) (94).

Central adiposity directly correlates with CVD risk (96, 97). Increase in central adiposity was able to alter HDL subclasses distribution, but overall HDL-C levels seemed not affected by this parameter (98). Obesity also affects HDL composition, function and subclasses distribution (99, 100). Obesity induces, most prominently in women compared to men, a pro-atherogenic dyslipidemia characterized by increased LDL and TG and reduced HDL-C, ApoA-I and ApoA-II levels. We and others showed that in morbidly severe obese patients bariatric surgery restores HDL endothelial-protective properties by modulating HDL composition (101–104). Bariatric surgery improves CV morbidity and mortality regardless of sex and gender (105, 106). Indeed, in a small patient cohort, we also showed after Roux-en-Y gastric bypass similar benefits on HDL endothelial protective function for both sexes (103). Circulating HDL-C levels increased in our patients after RYGB in agreement with other studies (107) however concentrations usually remains well below cut offs (80–90 mg/dL; 2.06–2.33 mmol/L) that are associated with higher CVD risk (53, 108). Finally, it is worth to consider that, in the context of obesity and bariatric surgery, gender-dependent differences (e.g., differences between women and men in the perception of their body weight in relation to esthetic, health and therapeutic perspective) are very important and need to be appraised when evaluating study results and identifying gaps of existing knowledge (106).

## Insight into sex-dependent regulation of EC function

Women before menopause have lower risk of developing CHD and endothelial-protective properties of estrogens can be important contributors (Figure 1) (109). Aging-driven reduction of flow-mediated dilation appears at the age of menopause, more than a decade later than in men, in concomitance with the loss of circulating estrogens (110). Chronic treatment with estrogens improved endothelial-dependent vasodilation in both ovariectomized animal models and post-menopausal women (111–113). Moreover, clinical studies suggested that estradiol treatment was able to revert endothelial dysfunction in post-menopausal women with atherosclerotic, non-stenotic arteries by preventing acetylcholine-induced coronary vasospasm (114). At present however, controversies still exist on the cardio-protective effect of hormonal replacement therapy in post-menopausal women (115).

**Figure 2:**
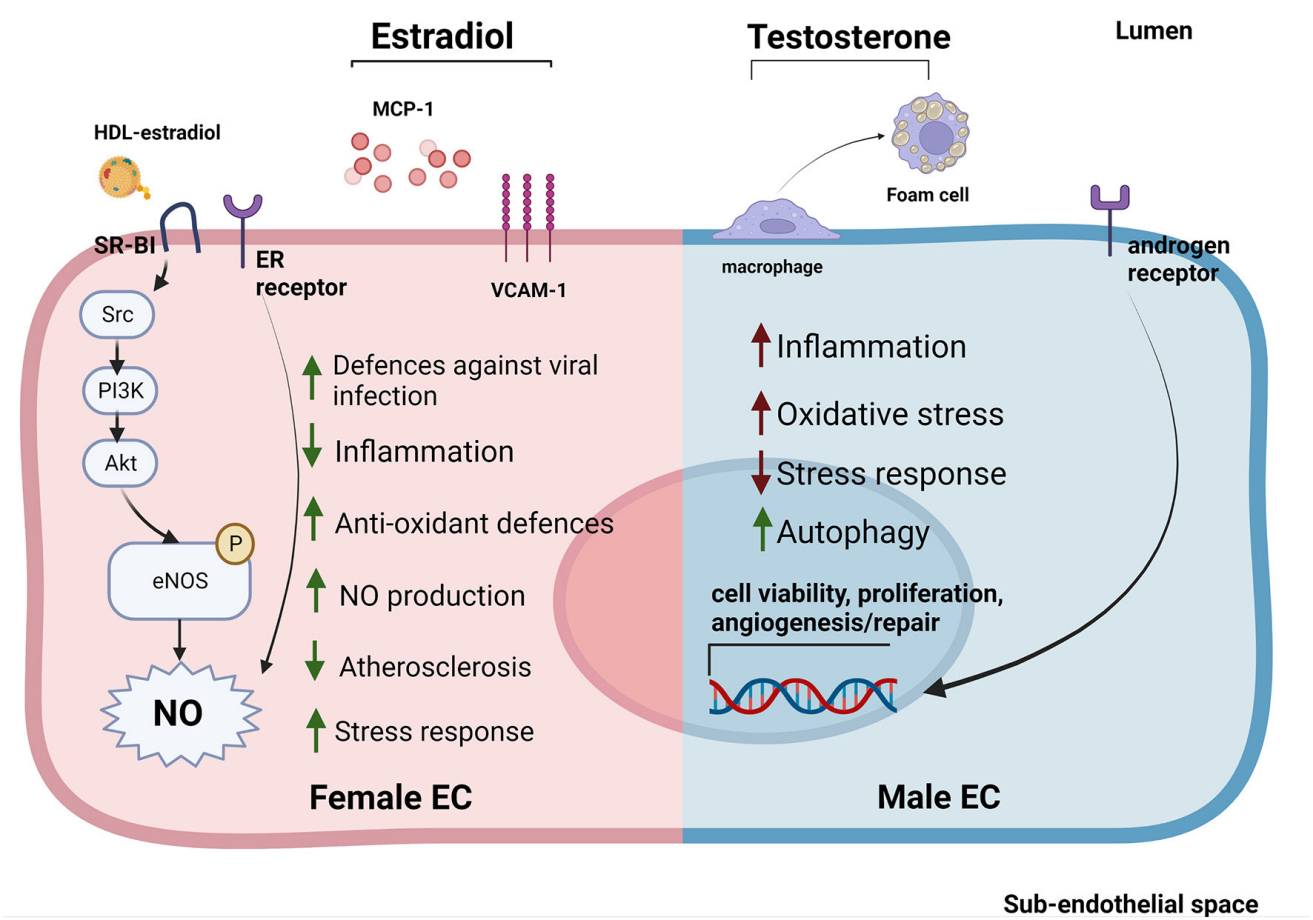
Sex-specific gene expression patterns and sexual hormones influence EC response to stimuli and EC-HDL cross talk. ECs derived from female donors **(left)** display reduced sensibility to stress and inflammation thanks to the activation of sex-specific pathways involved in stress response. Estradiol is able to reduce inflammation by reducing MCP-1 release and VCAM-1 expression. Furthermore, estradiol bound to HDLs is able to strongly activate SR-BI pathway increasing the NO production (53, 114, 116, 117). On the contrary, ECs derived from male donors **(right)** display a stronger susceptibility to inflammation, increased levels of oxidative stress and autophagy (118–120). Testosterone also contributes to create a pro-inflammatory environment by favoring the transformation of macrophages into foam cells (121). Estrogens stimulate NO production in both sexes through the activation of ER receptors (114). In contrast, androgen receptors are able to increase the expression of genes involved in cell viability, proliferation and angiogenesis/repair both in men and women ECs (122).

At the molecular level, estrogens can stimulate ECs primarily through estrogen receptors (ERα and Erβ, GPER1) on EC surface. Activation of ERs increases eNOS activity and NO production, thus promoting EC-mediated vasodilation (Figure 2) (116, 123). EC glycocalyx protects ECs from second insults after trauma hemorrhagic shock (T/SH) (117). Recently, it has been shown that estrogen administration after T/SH protects the EC glycocalyx from degradation by regulating tPA and PAI-1 levels, making ECs more resistant to additional damages (124). Furthermore, estradiol signaling attenuated endothelial inflammatory response by reducing cytokine and chemokine release (such as monocytes chemoattractant protein 1 (MCP-1) and IL-8) as well as EC adhesion molecule expression (125, 126). Prolonged exposure to estradiol also created a new homeostatic status in which immune cells were potentiated and ECs were less sensible to pro-inflammatory stimuli and apoptosis (Figure 2) (109).

While the EC protective role of estrogens have been well established, the effect of androgens on ECs is still under debate. Monocytes binding to aortic ECs seemed to be higher in male than female rabbits with hypercholesterolemia, suggesting a correlation between androgen levels and EC inflammation. However, this sex-dependent difference was also evident in non-hypercholesterolemic rabbits (127). Testosterone has also been associated with impaired vascular function in women (122, 128). This could be due to the fact that, although testosterone production is 10 times higher in men compared to women, women may be more sensitive to this hormone (129). Indeed, several studies pointed out an important sex-independent role of androgen receptors in regulating EC viability, proliferation, and angiogenesis/repair likely *via* upregulation of the VEGF-A, cyclin A, and cyclin D1 expression (Figure 2) (121). Of note, abundance of testosterone in male mice may favor its conversion into estradiol mediated by aromatase P450, causing hyper-activation of ERs promoting atherosclerosis (130, 131).

An overall marker of early atherosclerosis is the transformation of macrophages into foam cells through intracellular lipid accumulation. Treatment with testosterone promoted foam cell formation in men (but not in women) by increasing lipid loading, thus contributing to the development of atherosclerosis (Figure 2) (132).

Interestingly, transgender men treated with testosterone for 12 weeks displayed increased leukocytes-endothelium interactions, expression of adhesion molecules on EC surface, pro-inflammatory cytokine release, decreased HDL-C levels and dyslipidemia (23, 26). Furthermore, the levels of polymorphonucleate adhesion to ECs in transgender men were similar to diabetic men with silent myocardial ischemia, which highlight the need of a closer monitoring of cardiovascular risk in these patients (26). Progesterone, instead, protected ECs after cerebrovascular occlusion in male rats (133) and has been associated with increased NO production in women (134). However, administration of synthetic progesterone analogs, such as medroxyprogesterone, correlated with increased risk of coronary disease and stroke in women under hormonal replacement therapy (115, 135). Indeed, it has been shown that estrogen or progesterone and its synthetic analogs differently affect plasma lipoproteins, in particular, estrogen increases whereas progesterone and its synthetic analogs decrease HDL-C concentrations (136). Accordingly, while 17beta-estradiol had no effects, progesterone and three synthetic analogs suppressed ApoA-I-mediated cellular cholesterol release from human fibroblasts resulting in generation of less HDL particles (137).

Sex is a key variable in vascular biology and in particular, EC function is influenced by sexual hormones, but also by chromosomes resulting in sex-specific differences in gene expression profile. Human umbilical vein endothelial cells (HUVECs) derived from females and males were found intrinsically different independently from their exposure to sexual hormones, implicating a role for genomic sexual dimorphisms in CV system (14, 138). Transcriptomic performed in HUVECs in boy-girl twins or in non-twin adult ECs showed that sex-differences were present either at birth and maintained throughout life or acquired over life (118). As expected, sex differences in adult EC transcriptome involved many genes influenced by estrogens or androgens. Interestingly, androgen and estrogen receptors were not differentially expressed in adult ECs. Intriguingly, half of the genes showing sex-specific differences in HUVECs were sex chromosomal genes. Moreover, coronary artery disease targets (derived using multiple genome-wide association studies) were also enriched in the gene set showing sex difference in HUVECs, making possible to speculate about sex differences in CAD rooted in differential gene expression in ECs already at birth (118). Gene hallmark analysis showed increased expression of genes involved in endothelial to mesenchymal transition, NF-kB pathway and hypoxia in females, while increased expression in MYC targets, oxidative phosphorylation and mTOR pathway were reported in males (118). Other studies reported target-specific differences comparing male and female non-twin HUVECs, which may contribute to sex differences between males and females in endothelial function. Higher cell proliferation, migratory properties and endothelial NO synthase expression were observed in female HUVECs, while in the male cells beclin-1 and the LC3-II/LC3-I ratio, two widely accepted markers of autophagy, were higher (119). Notably, cellular size, shape as well as mRNA and protein expression of estrogen and androgen receptors were similar among sexes (119). Proteomic analysis of the secretome of serum-deprived HUVECs isolated from healthy female and male newborns revealed higher expression of proteins involved in cellular response to stress (e.g., several members of Annexin and Heat Shock Protein families) and apoptosis (e.g., PTX3) in male cells (120). These results are in agreement with reports obtained in different cells (e.g., neurons or cardiomyocytes) challenged with stressor stimuli and overall suggest lower resistance to oxidative stress and higher propensity of male cells to undergo apoptosis. On the contrary, female neurons/cardiomyocytes may be more resistant to oxidative stress with a pro-autophagy predisposition (139, 140); the latter characteristic will need to be further investigated in ECs as the above mentioned study on HUVEC transcriptome in boy-girls twins shows a male and not a female pro-autophagy gene signature (118).

Female HUVECs showed a stronger transcriptional response after shear stress exposure compared to male cells involving, for instance, upregulation of genes such as eNOS, heme-oxygenase 1 (HO-1) downregulation of NADPH oxidase 4 (Nox 4), endothelin-1 (ET-1) or vascular cell adhesion molecule 1 (VCAM-1), the latter downregulated by 22.2-fold in female vs only 3.5-fold in males (141).

Regarding EC energy supply, similar baseline ratios of glycolysis vs. mitochondrial respiration were observed in HUVECs obtained from male/female twins, but female cells performed better under starvation or under VEGF stimulation with higher ATP and metabolite levels compared to male cells, suggesting a more flexible modulation of energy production in females (120, 142).

Further studies will need to elucidate whether the described higher adaptability of female ECs to stress may confer them protection against CVD risk. Conversely, a stronger transcriptional response in female ECs might, in specific cases, favor disease onset and progression (e.g., in the context of the higher prevalence of autoimmune diseases in the female population) (143–145).

Collectively, increasing evidence highlights the presence of sex dependent differences in ECs at different stages of life. However, there are very few studies in adult ECs (i.e., HAECs) compared to the studies in HUVECs, which makes difficult to adequately investigate or compare changes in EC gene expression acquired later in life. Moreover, it is important to consider sex as a crucial biological variable not only in cardiovascular clinical research but also in experimental studies on EC biology to increase the quality and translational value of results.

## Insights into sex-dependent differences in HDL and EC crosstalk

### Lifestyle and CVD: Sex-dependent differences

Smoking, alcohol consumption, diet and exercise are modifiable CVD risk factors (Table 1).

**Table 1:** Comparative description of the effect of lifestyle habits on HDLs and ECs in men and women.

	**Men**	**Women**
Smoking	Increases CHD risk (147, 148)	Increases CHD risk (147, 148)
	Induces EC dysfunction (147, 148)	Induces EC dysfunction (147, 148)
	Reduces HDL number and functionality [147, 148]	Reduces HDL number and functionality (147, 148)
	Promotes inflammation (147, 148)	Promotes inflammation (147, 148)
	Reduces EPCs number (157)	Alters estrogen metabolism (151, 152)
		Increases risk of CVD and MI (152)
Alcohol	Increases HDL-C levels (162)	Increases HDL-C levels (162)
	Prevents EC activation and inflammation (170)	Reduces stroke risk (169)
		Increases overall mortality (169)
		Prevents EC activation and inflammation (170)
Diet	Mediterranean diet: reduces small dense LDL and increases medium LDL, reduces EC inflammation and oxidative stress (174, 175)	Mediterranean diet: reduces medium dense LDL and increases small LDL, reduces EC inflammation and oxidative stress. Increases eNOS activity and reduces CVD risk. (174, 175, 192)
		Dairy diet: Reduces insulin sensitivity (176, 177)
Physical activity	Prevents EC dysfunction and atherosclerosis (194, 195)	Prevents EC dysfunction and atherosclerosis (194, 195)
	Increases HDL-C levels (198, 199)	Increases HDL-C levels (198, 199)

CHD, Coronary Heart Disease; EC, Endothelial Cells; HDL, High-Density Lipoproteins; HDL-C, HDL-Cholesterol; CVD, Cardiovascular Disease; MI, Myocardial Infarction; LDL, Low-Density Lipoproteins; eNOS, endothelial Nitric Oxide Synthase; EPCs, Endothelial Precursor Cells.

The number of smoked cigarettes positively correlated with increased CHD risk in both sexes. In fact, smoking induced endothelial dysfunction and damage, increasing lipid oxidation, decreasing HDL, and promoting inflammation, and a pro-thrombotic state (146, 147). Furthermore, a worse lipid profile characterized by increased ApoB and reduced ApoA-I and ApoA-II was reported in smokers compared to non-smokers independently from their sex (148). Interestingly, smoking was reported as a stronger risk factor for CVD in women than in men accordingly to the Finnmark Study (149). This could be partially attributed to the ability of smoking to alter estradiol metabolism leading to the formation of inactive catechols (150, 151), thus inhibiting estradiol vaso-protective properties. Moreover, exposure to passive smoking from birth was associated with reduced HDL-C levels in adolescent girls but not in boys (152). The anti-estrogenic effect of smoking positively correlates with increased CHD risk and strong reduction in HDL-C levels in young compared to older women and men (150, 153). The evidence that ex-smokers had higher HDL-C levels compared to smokers of both sexes further confirm these results (154). Furthermore, the Copenhagen City Heart Study reported that smoking women had 9.4 higher risk of myocardial infarction compared to non-smoking women, while the risk score was only 2.9 times higher in smoking men compared to non-smokers (155). On the contrary, reduced levels of endothelial progenitor cells (EPCs) have been reported in men compared to women. Smoking further decreased EPCs in men, while no difference was found between smoking and non-smoking women. In this case, sex-differences on the effect of smoking were mostly attributed to a protective effect of estradiol on EPCs (156).

Low to moderate alcohol consumption did not affect CVD risk in both sexes (157). The CoLaus Study reported no differences in expression of HDL-related genes (i.e., ABCA1, APOE5, CETP, hepatic lipase and lipoprotein lipase) based on alcohol consume in a sex-mixed pool of Caucasian patients (158). CHD risk could perhaps vary depending on the ethnicity of the patients. The Atherosclerosis Risk in Communities (ARIC) study reported reduced CHD in whites but increased disease in black alcohol consumer men independently from levels of alcohol consumed (159). This was partially attributed to different hepatic gene variants and expression levels (i.e., CETP, hepatic lipase, LPL, and PON1) between these two ethnicities (159, 160). Meta-analysis data suggested that HDL-C levels increased an average of 0.06 mmol/L per 23 g/day of alcohol consumed (161). The increase of HDL-C levels in alcohol consumers have been attributed to enhanced HDL production (hepatic and extra-hepatic), decreased CETP activity and lower HDL-C clearance (162). However, this increase is strongly influenced by alcohol-gene interactions (163). As an example, men and post-menopausal women carrying the homodimeric γ2 variant of the ADHIC gene had a slower rate of alcohol clearance, which was associated with elevated HDL-C levels (164, 165). It is worth specifying that sex differences in alcohol consumption are difficult to detect, since generally women can tolerate lesser amount of alcohol due to their sex-specific absorption, body fat/water ratio, reduced levels of enzymes involved in alcohol metabolism and glomerular filtration rate (166). Furthermore, studies in the general population indicated that among all alcohol consumers/abusers, only 1/3 were women (167). Nevertheless, moderate alcohol consumption was associated with lower risk of stroke in women compare to men but also with a 10% increased risk of overall mortality (168).

Alcohol has also a direct effect on ECs. Indeed, moderate levels of alcohol were able to prevent endothelial activation and pro-inflammatory cytokine release in human coronary artery ECs stimulated with the pro-inflammatory SAA (169). Furthermore, a reduction in monocyte adhesion to TNF-α-stimulated ECs was reported in moderate alcohol consumer men compared to non-consumers (170). On the other hand, heavy alcohol consumption (both measured and self-reported) was associated with increased circulating vascular adhesion molecules (i.e., E-selectin, intracellular adhesion molecule 1 (ICAM-1) and VCAM-1) and reduced flow-mediated dilation in sex-mixed cohorts of patients, independently of alcoholic liver disease (171, 172).

Diet also affects women and men in a different manner. Mediterranean diet was able to reduce total cholesterol, LDL-C, ApoB and ApoA-1 plasma levels in both sexes. However, men under Mediterranean diet experienced a reduction in small dense LDL and an increase in medium LDL, while the opposite trend was observed in women (173, 174). Furthermore, a comparison between red meat and dairy diet highlighted a reduction in insulin sensitivity in women following the dairy diet. Instead, no differences between the two diets were reported in men (175, 176). ABCA1 is one of the most sex-influenced gene in the liver and its expression is higher in females (177). Indeed, estrogen levels and dietary components were able to regulate ABCA1 expression in macrophages, leukocytes and liver in human and rodents, increasing ApoE-positive HDL particles and improving cholesterol efflux (178–181). Among all the genetic variants, ABCA1/R230C was associated with low HDL-C (182). It has been shown that dietary macronutrient proportions regulated the effect of ABCA1/R230C in premenopausal women by directly interacting with ABCA1 gene (183). In particular, metabolically unfavorable pattern was found in ABCA1/R230C premenopausal women following high carbohydrates and low fat diet, while the opposite pattern was found in women following high fat and low carbohydrates diet (183).

On the contrary, lowering dietary fat intake was able to restore HDL functionality (in particular HDL-CEC) in hypercholesterolemic female pigs by reducing cholesterol plasma levels (184).

There is some evidence that diet could directly affect EC function. Transitory disruption of endothelial function and reduction in vasorelaxation have been reported after acute administration of high-fat meal, in concomitance with increased triglyceride-rich lipoproteins in plasma (185, 186). On the contrary, chronic consumption of low-fat diets (i.e., Mediterranean diet) was associated with improved endothelial function and reduced markers of endothelial activation in plasma in men (187–189), most likely through changes in cholesterol metabolism and the presence of oleate and decosahexanoic acid, which were able to reduce pro-inflammatory molecule expression and monocyte adhesion in ECs *in vitro* (190). Same results were shown also in women. Indeed, a pilot study demonstrated that specific components of Mediterranean diet (i.e., legumes, red meat, and overall proteins) were associated with reduced inflammation and oxidative stress, increased eNOS activity and reduced CVD risk in a cohort of ethnically mixed women (191).

Physical activity is well known as protective factor against CVD (192). Evidence showed that physical activity was able to slow down EC dysfunction and atherosclerosis progression in both sexes (193, 194). However, how physical activity differently influence CVD risk in women and men is still under debate. A systematic review focused on physical activity and stroke incidence claiming that, among all the analyzed studies, 35% of them reported a strong association between physical activity and stroke incidence in women, while the same correlation in men was evident only in few studies (195). Even so, the Framingham Study reported that physical activity conferred protection against stroke in men, but not in women (196).

The correlation between physical activity and reduction in CVD risk could be partially attributed to increased HDL-C levels in physically active individuals compared to sedentary ones. However, the increase in HDL-C seemed to be significant only when a threshold volume of physical activity was reached (197, 198). Even if the threshold level has not been accurately and systematically estimated yet, epidemiological and cross-sectional studies suggested that the threshold value was 1,500 kcal/week in men and 1,200 kcal/week in women independently from their menopausal status (199–201).

### Reverse cholesterol transport

The best-known property of HDL is RCT, which consists in the ability of HDL to accept excess cholesterol from peripheral cells, in particular macrophages, and transport it to the liver for excretion or re-utilization. RCT is considered the most important anti-atherogenic function of HDLs. Components of cholesterol efflux include the passive diffusion of cholesterol from cells as well as the active cellular cholesterol transfer by ABCA1, ABCG1, and SR-BI. In this context, ECs may represent a potential barrier to HDL in reaching macrophages within the vessel wall. However, HDL and lipid-free ApoA-I are able to cross intact aortic EC monolayers from the apical to the basolateral compartment in a transcytosis process, involving ABCG1 and SR-BI (202). Contrary to other cells that form the atherosclerotic plaque (smooth muscle cells and macrophages), ECs do not accumulate cholesterol (203) and have a strong ability to efflux cellular cholesterol to HDLs independently of ABCA1, ABCG1, and SR-BI expression or activity (204). On the other hand, it has been shown that in conditions of hyperlipidemia ECs can metabolize LDLs into cholesterol crystals, which accumulate intracellularly and confer a foam cell-like morphology to ECs (205).

HDLs derived from healthy normolipidemic men and women had different RCT capacities due to the activation of sex-specific mechanisms for cholesterol efflux (206). The rs1799837 (APOA1) and rs1800588 (LIPC) SNP variants represented the major determinants of HDL cholesterol efflux capacity in women, while rs2230806 (ABCA1) and rs5082 (APOAII) variants were key determinants in men (207). Furthermore, serum isolated from women displayed an enrichment in large-HDL-particles (L-HDL-P) and increased capacity to mediate cholesterol efflux through SR-BI receptors. On the other hand, serum isolated from men showed increased preβ-HDL particles and cholesterol efflux through ABCA1 receptors (206). Both low and high HDL-C levels were associated with reduced free cholesterol transfer on HDLs in both sexes, especially in women, in patients with acute myocardial infarction and Tangier disease (208).

These findings not only suggest that HDL composition differs in men compared to women, but also that these differences in HDL pool may have an impact in their ability to stimulate cholesterol efflux. Estradiol levels, in fact, positively correlated with HDL cholesterol efflux capacity (HDL-CEC) from macrophages in pre-menopausal women and were associated with increased concentration of L-HDL-P and lower concentration of small-HDL-particles (S-HDL-P) (209). However, HDL-CEC decreased in transgender women (men to women) under estradiol hormone therapy, suggesting that reduction of testosterone and increase in estradiol may act synergistically in reducing HDL-CEC (24). This hypothesis is in line with the evidence that testosterone deprivation in men increased HDL-C levels but not HDL-CEC, while estradiol treatment had the opposite effect (210). The correlation between androgen levels and CVD in men is controversial. Low levels of androgens were associated to increased CVD in older men (211). On the other hand, testosterone administration in hypogonadal men can blunt EC-mediated vasorelaxation (212, 213). Thus, age and type of androgen used are important factors to be considered.

### Inflammation

Increased expression levels of specific adhesion molecules—such as VCAM-1 and ICAM-1 and E-selectin—are a well-recognized marker of EC inflammation and oxidative stress. HDL particle concentration is inversely correlated with the expression of cellular adhesion molecules, as well as inflammatory mediators C-reactive protein (CRP) and TNF-α. The underlying mechanism can be partly attributed to HDL-associated sphingosine 1 phosphate (S1P). S1P signaling through S1P receptor has been shown to protect against TNF-α-induced monocyte binding to ECs preventing the activation of NF-kB and c-Jun pathways as well as reducing the secretion of pro-inflammatory chemokines (214).

OxLDLs can induce MCP-1, which is involved in the recruitment of monocytes into the sub-endothelial space and their differentiation into foam cells. It is an important inflammatory process in the initial stages of atherosclerosis (215). *In vitro* and *in vivo* experimental studies suggested that HDL associated-PON1 inhibited LDL-oxidation by catalyzing the breakdown of oxidized phospholipids, thus abolishing the production of pro-inflammatory cytokines (MCP-1, IL-8 and macrophage colony stimulating factor) from ECs (216–220).

HDLs are also able to transfer microRNA to ECs (221). It was shown that HDL-transferred microRNA-223 directly targeted ICAM-1 gene at 3'UTR sites suppressing gene expression and function in HUVECs stimulated with TNF-α thus reducing leukocyte adhesion. Regulation of TNFα-induced ICAM-1 expression by HDLs was not found in fibroblasts, suggesting a specific miR-223 delivery on ECs (222–224).

HDL-induced NO production also plays an important role in the reduction of EC inflammation. The induction of PI3K-Akt-eNOS signaling mediated by the binding of ApoA-1 and SR-B1 up-regulates cyclooxygenase (COX-2) expression and prostaglandin I2 (PGI_2_) release in ECs (225). PGI_2_ is a potent inhibitor of inflammation, which limits immune cell proliferation as well as inhibits platelet aggregation, thus affecting smooth muscle relaxation and vasodilation (226).

HDL-C levels also correlated with risk of infection. Similarly to what was showed regarding all-cause mortality risk (3, 4), both low and high levels of HDL-C were associated with increased risk of infection (227). The increased risk of infection associated with low levels of HDL-C could be in part due to the loss of leucopoietic control and immune cell modulation from HDLs (228, 229). The mechanism in case of high levels of HDL-C is less clear, but particular genetic mutations associated with increased HDL levels may also affect disease susceptibility. For instance, in case of LIPC and SCARB1 (encoding for hepatic lipase and SR-BI, respectively), whose mutations were associated with increased risk of CAD (46, 230). Interestingly, several SNP variants located in the promoter and intron 1 of LIPC gene were associated with changes in HDL-C levels in women but not in men (231). SCARB1 rs5888 SNP variant, instead, has been associated with a greater reduction in total cholesterol, LDL-C and ApoB in women treated with atorvastatin compared to men in patients with acute coronary syndrome (232).

Peri-menopausal and menopausal women had increased levels of TNF-α, CRP, TG and LDL compared to their pre-menopausal counterparts, which may underline a reduction on HDL functionality driven by the collapse in estrogen levels (233).

HDL anti-inflammatory properties are impaired or lost in chronic inflammatory conditions. Concomitantly with a decrease of HDL particle levels also changes in the structure and function of HDLs are observed. Patients with chronic inflammatory disorders, including rheumatoid arthritis, systemic lupus erythematosus, and psoriasis, which are associated with an increased risk of atherosclerotic CVD, exhibit a consistent decrease in HDL particles and ApoA-1 levels and HDL vaso-protective properties in a sex-independent manner (234–236). However, in other diseases as in the antiphospholipid syndrome, studies reporting a strong reduction in HDL functionality were conducted comparing only affected and healthy women (237). Thus, it will be important to further elucidate with sex-matched comparisons whether or not men and women HDLs are similarly affected also in those immune-inflammatory diseases.

Interestingly, it has been recently shown that HDL-C and ApoA-I levels measured before SARS-CoV-2 infection negatively correlated with COVID-19 mortality and hospitalization, independently of age, sex, comorbidities, or statin treatment (238–240). Furthermore, HDL cargo was profoundly altered in severe COVID-19 patients, with increased abundance of SAA-1 and−2, SFTPB, ApoF, and inter-alpha-trypsin inhibitor heavy chain H4 (241). These findings are corroborated by the evidence that treatment with reconstituted HDLs in COVID-19 patients reduced SAA-1, SFTPB, and ApoF in HDLs (242). Of note, men with COVID-19 were more prone to develop into the severe condition and die compared to women (Figure 2) (243, 244). Indeed, no striking differences were found between pre- and post-menopausal women, suggesting that reduction in female mortality may be independent from estrogen levels (243, 244). *In vitro* experiments conducted on ECs exposed to SARS-CoV-2 S1 spike protein showed a significant increase in the overall inflammatory status in cells treated with androgens (245). Recent findings showed that male sex clinical-biological characteristics, rather than male gender-related differences (i.e., pertaining to the socio-economic sphere, such as education) were independently associated with intensive care unit admission, invasive ventilation, and/or death in COVID-19 (246).

### Anti-apoptotic and anti-oxidative properties

EC homeostasis relies on the balance between pro- and anti-apoptotic stimuli coming from bloodstream and neighboring cells (247, 248). Once the balance is disrupted, (pro)-apoptotic ECs favor platelet aggregation and coagulation, creating a pro-atherogenic environment (249, 250). OxLDL can promote EC apoptosis by increasing intracellular Ca^2+^ levels (251–253), favoring the onset and progression of CVD (114). In contrast to LDLs, HDLs protect ECs from apoptosis by preserving mitochondrial integrity and inhibiting the activation of the caspase-downstream cascade (254–256). Indeed, HDLs isolated from a mixed sex pool of healthy donors was able to reduce EC apoptosis both *in vivo* and *in vitro*, while sex-matched HDLs isolated from CAD patients showed opposite results (257).

In particular, HDL particles containing ApoA-I seemed to be more cytoprotective than other HDL subclasses (258, 259).

Moreover EPCs can quickly differentiate into mature ECs to rescue vascular integrity in conditions of high cell turnover (260). HDLs can promote endothelial repair by increasing EPC number and function in male mice (261, 262).

In addition to their anti-apoptotic properties, HDLs can also reduce oxidative stress. PON-1 is an accessory protein of HDL that, in coordination with ApoA-I, protect lipoproteins, ECs and intimal macrophages from oxidative insults by hydrolyzing lipo-lactones (263–266). High levels of oxidative stress can increase HDL lipid peroxide loading, decreasing their protective activity against LDL oxidation (267, 268). Significant differences in HDL peroxide levels between men and women have been reported as a readout of sex-specific reduction in HDL anti-oxidative functions in men (269).

Decreased HDL anti-oxidative properties driven by high glycemic burden have also been reported in both type 1 and type 2 diabetic men and post-menopausal women (270, 271). In this context, it has been shown that PON-1 activity was more strongly impaired in T2D women compared to men (272).

Furthermore, women with hypertension, metabolic syndrome or peri-menopause not only had higher levels of oxLDLs compared to healthy middle-age or pre-menopausal women, but also reduced defenses against oxLDLs. However, the contribution of estrogens in this context is still unclear (273–275).

HDLs can serve as carriers for other molecules, for instance estrogens. It has been shown that the binding of estrogens with HDLs increases their anti-oxidative properties due to estrogen esterification performed by LCAT (276). Indeed, LCAT was able to esterify HDL-bounded E2. Esterified E2 was then transferred from HDL to LDL thanks to CETP (276). Incubation experiments demonstrated that E2 esterification and further association with LDL was able to increase LDL resistance to oxidation (276, 277). On the contrary, hyperandrogenism is associated with increased oxidative stress and reduced HDL anti-oxidative functionality in women (278, 279). To the best of our knowledge HDLs have not been reported as carriers for androgens.

### HDL-mediated endothelial NO-production

Several mechanisms account for the endothelial NO-stimulating capacity of HDLs. In cultured ECs, HDLs directly activate the production and release of NO by binding of ApoA-I to SR-BI, leading to increased intracellular ceramide levels and phosphorylation of endothelial NO-synthase (eNOS) (280). Cholesterol efflux from ECs to HDLs *via* ABCA1 also promotes NO synthesis by modulating cholesterol-binding protein caveolin-1 and eNOS (281). Mechanistically, the binding of HDLs to SR-B1 leads to tyrosine kinase Src-mediated activation of phosphoinositide 3-kinase (PI3K), which in turn stimulates Akt and Erk pathways. The activation of Akt directly stimulates eNOS by phosphorylation at Ser-1179 (282) (see Figure 2).

Estradiol is able to induce rapid arterial vasodilation by stimulating eNOS activity by acting *via* ERs (18). HDLs isolated from female mice and healthy women (but not male mice or men) were able to enhance NO production. In addition to ERs, estradiol bound to HDLs was also able to enhance eNOS activity through the activation of SR-BI receptors (Figure 2) (283).

Specific lipid and protein components of HDLs have a strong impact on NO production. S1P is a lipid carried mainly on apoM-containing HDLs (>50% of circulating S1P). S1P-apoM HDLs are involved in persistent activation of Akt/eNOS pathway, thus leading to NO dependent vasodilation through S1P receptor stimulation (284–286). Decreased levels of ApoM/HDL correlated with increased CVD risk and have been reported in type 2 diabetes in both sexes and in women (but not in men) with type 1 diabetes (287–289).

Reduced HDL-associated PON-1 activity leads to the activation of endothelial lectin-like oxidized LDL receptor (LOX-1) and PKCαII, thus inhibiting the activity of eNOS (290). Inflammation and metabolic syndromes can also drastically affect the ability of HDLs to stimulate NO production (291, 292), thus promoting atherosclerosis (293).

## Conclusions

Sex hormones and sex-specific gene expression are important although still incompletely understood determinants in the regulation of HDL and EC cross talk and their contribution to cardiovascular health and disease. Despite increasing evidence pointing out that sex-origin of cultured cells and in particular ECs can strongly affect scientific results (294–296), most of the articles do not report any information about the sex of the cells or of the animals used in their experiments. The use of sex-mixed cohorts of patients or imbalanced number of women and men during clinical trials also represents a potential source of bias. As highlighted in this Review, it is of foremost importance to always consider the influence of sex as biological variable in each step of research and to clearly report and analyze this information.

## Author contributions

ED, AJ, and EO wrote the manuscript. ED designed the figures under BioRender common creative license. All authors contributed to the article and approved the submitted version.

## Funding

This research was funded by the Swiss National Science Foundation (SNSF), grant number PRIMA: PR00P3_179861/1 and the Swiss Life Foundation, the Alfred and Annemarie von Sick Grants for Translational and Clinical Research Cardiology and Oncology, the Heubergstiftung and the Swiss Heart Foundation, Switzerland all to EO.

## Conflict of interest

The authors declare that the research was conducted in the absence of any commercial or financial relationships that could be construed as a potential conflict of interest.

## Publisher's note

All claims expressed in this article are solely those of the authors and do not necessarily represent those of their affiliated organizations, or those of the publisher, the editors and the reviewers. Any product that may be evaluated in this article, or claim that may be made by its manufacturer, is not guaranteed or endorsed by the publisher.
